# Switching to Regular Diet Partially Resolves Liver Fibrosis Induced by High-Fat, High-Cholesterol Diet in Mice

**DOI:** 10.3390/nu14020386

**Published:** 2022-01-17

**Authors:** Muhammad Farooq, Huma Hameed, Marie-Thérèse Dimanche-Boitrel, Claire Piquet-Pellorce, Michel Samson, Jacques Le Seyec

**Affiliations:** 1 Inserm, EHESP, IRSET (Institut de Recherche en Santé, Environnement et Travail)-UMR_S 1085, Univ Rennes, F-35000 Rennes, France; muhammad.farooq@uvas.edu.pk (M.F.); huma4748@gmail.com (H.H.); marie-therese.boitrel@univ-rennes1.fr (M.-T.D.-B.); claire.piquet-pellorce@univ-rennes1.fr (C.P.-P.); jacques.leseyec@univ-rennes1.fr (J.L.S.); 2 Department of Clinical Sciences, College of Veterinary and Animal Sciences, Jhang 35200, Pakistan

**Keywords:** diet, liver, NASH, fibrosis, reversibility

## Abstract

The globally prevalent disease, non-alcoholic steatohepatitis (NASH), is characterized by a steatotic and inflammatory liver. In NASH patients, tissue repair mechanisms, activated by the presence of chronic liver damage, lead to the progressive onset of hepatic fibrosis. This scar symptom is a key prognostic risk factor for liver-related morbidity and mortality. Conflicting reports discuss the efficiency of dietary interventions on the reversibility of advanced fibrosis established during NASH. In the present study, the effect of dietary interventions was investigated in the outcome of the fibrosis settled in livers of C57BL/6J mice on a high-fat, high-cholesterol diet (HFHCD) for 5 or 12 consecutive weeks. Various clinico-pathological investigations, including a histological analysis of the liver, measurement of plasma transaminases, steatosis and fibrosis, were performed. To assess the effectiveness of the dietary intervention on established symptoms, diseased mice were returned to a standard diet (SD) for 4 or 12 weeks. This food management resulted in a drastic reduction in steatosis, liver injuries, inflammatory markers, hepatomegaly and oxidative stress and a gradual improvement in the fibrotic state of the liver tissue. In conclusion, our results demonstrated that dietary intervention can partially reverse liver fibrosis induced by HFHCD feeding.

## 1. Introduction

Non-alcoholic fatty liver disease (NAFLD) varies from simple steatosis without hepatocellular injury (NAFL) to the NASH aggressive form [1,2]. NAFL is reversible and characterized by at least 5% of liver steatosis. It can progress into NASH, which is characterized by inflammation, oxidative stress and cell death with or without liver fibrosis [3,4]. NASH can further progress into liver cirrhosis and hepatocellular carcinoma (HCC). Currently, NASH is the second leading cause of liver transplantation and is predicted to be the leading cause in the next decade [5]. This affection is also associated with insulin resistance and obesity [1,2]. The pathogenesis of NASH is not fully elucidated. In 2016, Buzzetti et al. proposed a multiple-hit hypothesis to better understand the development mechanism [6]. Thus, lipotoxicity, in parallel with other dietary and genetic factors, adipose tissue dysfunction and gut microbiome regulate cell death and inflammation, which leads to the development of NASH. In this context, the activated hepatic stellate cells (HSCs) are responsible for the gradual onset of fibrosis.

Collagen, laminin, proteoglycans, fibronectin and matricellular proteins are part of the extracellular matrix (ECM) [7]. In healthy livers, there is a balance between ECM synthesis and degradation. Thus, the abundance of matrix proteins is regulated by proteases from the family of matrix metalloproteinases (MMPs) and of a disintegrin and metalloproteinases (ADAMs), themselves under the control of the tissue inhibitors of MMPs (TIMPs) [8]. The disruption of the intricate balance between synthesis and degradation results in an increased deposition of ECM at the origin of liver fibrosis. When the causative agent of liver damage disappears, fibrosis can be reversed, meaning that proteases degrade ECM [9]. However, under certain conditions, fibrosis only partially resolves in some patients without preventing the onset of severe complications, such as HCC [10,11,12].

To evaluate the reversibility of liver fibrosis, different rodent models have already been used. The primary model was based on repeated injections of carbon tetrachloride (CCl4). Once chronic exposure to the hepatotoxic agent is stopped, hepatic fibrosis resolves [13]. However, this chemical-induced model does not reflect NASH pathophysiology since it does not take into account the nutritional and metabolic parameters of the human disease. Different animal experimental models of NASH have been established in an attempt to reproduce this complex pathology and to study the potential for symptom reversion and, in particular, fibrosis. Thus, investigations were conducted to assess the dietary modifications on fibrosis reversibility in models by either switching a Western diet (WD) to a non-purified and purified chow diet, a methionine choline-deficient diet (MCD) to a methionine choline control diet (CD) or a choline-deficient L-amino acid-defined diet (CDAA) to a choline-supplemented diet [14,15,16]. Even if some models induce only weak fibrosis, the role of diet-switching in resolving fibrosis appears controversial [14,15,16,17]. Previously, we have demonstrated that the high-fat, high-cholesterol diet (HFHCD) feeding of mice induces NASH, characterized by hepatocyte ballooning, liver inflammation, elevated transaminases and extensive chicken wire liver fibrosis, a key finding in human liver fibrosis during NASH [18]. In this model, severe hepatic fibrosis can reach up to 20% of the liver tissue [18,19]. In the present study, we sought to investigate the reversibility of the histopathological parameters following the establishment of severe NASH promoted by HFHCD consumption by the cessation of HFHCD feeding and switching to a normal chow diet.

## 2. Materials and Methods

### 2.1. Animal Model

Adult C57BL/6J male mice (10 to 12 weeks old) were fed ad libitum with a standard diet (SD, #2016, Teklad Diet, Envigo, Gannat, France) or a high-fat, high-cholesterol diet (HFHCD, #9G21, LabDiet, St. Louis, MO, USA). HFHCD is an HFD supplemented with cholesterol and contains a high percentage of saturated fatty acid. After a period of 5 or 12 weeks on HFHCD, the mice eventually returned to an SD for 4 or 12 weeks (*n* = 7 in each group). A schematic diagram of the experimental design and diet composition were shown in supplementary data (Figure S1 and Tables S1 and S2).

### 2.2. Histopathological and Biochemical Studies

The mouse liver was collected after euthanasia. Liver fragments were fixed in 4% paraformaldehyde and embedded in paraffin for immunohistochemistry. For histopathology, 4 µm tissue sections were stained, either with hematoxylin and eosin (H&E) to study liver injury, with Sirius red for fibrosis analysis or with Oil Red O (ORO) with hematoxylin counterstaining to assess steatosis. The proportions of Sirius-red-stained areas or of ORO-labeled surfaces were, respectively, determined using NIS-Elements software (Nikon) or HALO software (Indica Labs, Albuquerque, NM, USA). Plasma alanine (ALT) and aspartate (AST) transaminases were measured according to the International Federation of Clinical Chemistry and Laboratory Medicine (IFCC) primary reference procedures using the Olympus AU2700 Chemistry Analyzer (Olympus Optical, Tokyo, Japan). Only the ALT data are depicted since those of the AST systematically followed the same variations.

### 2.3. Imaging by Second Harmonic Generation (SHG)

SHG microscopy is a nonlinear imaging technique based on the optical effect that is used to visualize the endogenous extracellular matrix components of liver tissue in a more sensitive and specific manner. Multiphoton imaging by second-harmonic generation (SHG) and two-photon excitation fluorescence (TPEF) was applied on paraformaldehyde-fixed and paraffin-embedded liver sections. The SHG imaging system was composed of a confocal TCS SP5 scanning head (Leica Microsystems, Mannheim, Germany) mounted on a DM IRE2 inverted microscope (Leica Microsystems) and equipped with a Mai Tai femtosecond laser (Spectra Physics, Santa Clara, CA, USA). A 60x water immersion objective (Olympus LUMFL 60 W × 1.1 NA) was used for applying a 10–20 mW of 820 nm excitation to the sample. The SHG signal was collected in the forward direction using a water immersion condenser (S1, NA ¼ 0.9–1.4; Leica Microsystems), and the TPEF was epi-collected in the backward direction. IRSP 715 bandpass and 410 nm infrared (IR) filters (10 nm full width at half-maximum, FWHM) were placed before the photomultiplier tube. Slides of liver samples were positioned on the fixed x, y stage of the microscope with light propagating in the z direction. The input polarization was chosen along the y axis and perpendicular to the main x axis of the liver tissue. Collagen was detected from the SHG channel by the Ostu’s automatic threshold method. The image processing was performed with ImageJ software (National Institutes of Health; http://rsb.info.nih.gov/ij/, accessed on 15 October 2021).

### 2.4. RNA Isolation and RT-qPCR

Total RNA was extracted from mice liver tissues using the NucleoSpin^®^ RNA kit (Macherey-Nagel, #740955, Hoerdt, France). First-strand cDNA was synthesized using the High-Capacity cDNA Reverse Transcription Kit (Applied Biosystems, #4368813, Foster City, CA, USA). Real-time quantitative PCR was performed using the double-strand-specific SYBR^®^ Green master mix (Applied Biosystems, #4367659, Villebon-sur-Yvette, France) on the CFX384 Touch Real-Time PCR Detection System (Biorad, Marnes-La-Coquette, France). Each measurement was performed in triplicate. The relative gene expression was normalized against the 18 S gene expression. Samples from healthy mice only fed an SD served as references for mRNA expression (control mRNA level was arbitrarily set at 1). All primer sequences are depicted in Table S3 in the supporting information.

### 2.5. Statistical Analysis

Data were expressed as means ± SEM for all mice treated similarly. Mean differences between experimental groups were assessed using the non-parametric Kruskal–Wallis test. For pairwise comparison, post hoc Dunn’s test was applied. All statistical analysis was achieved with GraphPad Prism5 software. Significance is shown as follows: * *p* < 0.05, ** *p* < 0.01, *** *p* < 0.001 and **** *p* < 0.0001.

## 3. Results

### 3.1. HFHCD-Induced Liver Damage and Hepatomegaly Are Reversible after Switching to SD

Previously, we demonstrated that HFHCD feeding of mice induced NASH, mimicking the human disease [18].

**Figure 1:**
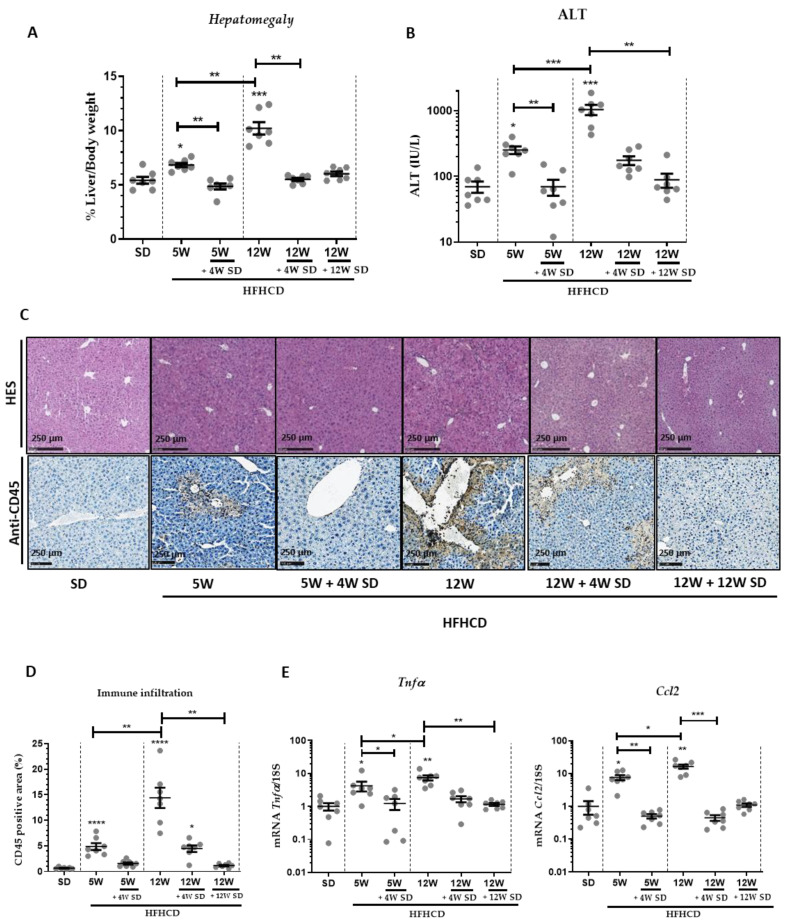
Dietary intervention resulted in significant changes in hepatomegaly, liver damage and inflammation. Six groups of seven male mice were given separate diet regimens of either a standard diet (SD) or a high-fat, high-cholesterol diet (HFHCD) for 5 or 12 weeks (W), with some groups returning to the SD on an additional period of 4 or 12 weeks. (**A**) Percentage of liver weight to body weight. (**B**) Level of ALT in plasma. (**C**) Hematoxylin and eosin (H&E; upper panels) and CD45 staining (lower panels) of liver sections. (**D**) Quantification of immune infiltrates measured from CD45 positive area. (**E**) Liver transcript expression of Tnf-α and Ccl2. For all graphs, each dot represents an individual, and error bars are expressed as means ±SEM (* *p* < 0.05, ** *p* < 0.01, *** *p* < 0.001 and **** *p* < 0.0001).

The HFHCD feeding caused characteristic symptoms of NASH, starting with the gradual induction of hepatomegaly. Indeed, this symptom appeared as early as 5 weeks of HFHCD and continued to worsen after an additional 7 weeks of the same diet (Figure 1A). However, this clinical manifestation completely and rapidly disappeared when mice were returned for 4 weeks to an SD. Similarly, HFHCD feeding promoted liver damage, as evidenced by the increase in serum ALT reaching, at the 12-week stage, high values for a chronic hepatitis model (1000 IU/L) (Figure 1B). Despite the severity of the liver damage, serum ALT levels returned to basal values after switching the diet to standard.

**Figure 2:**
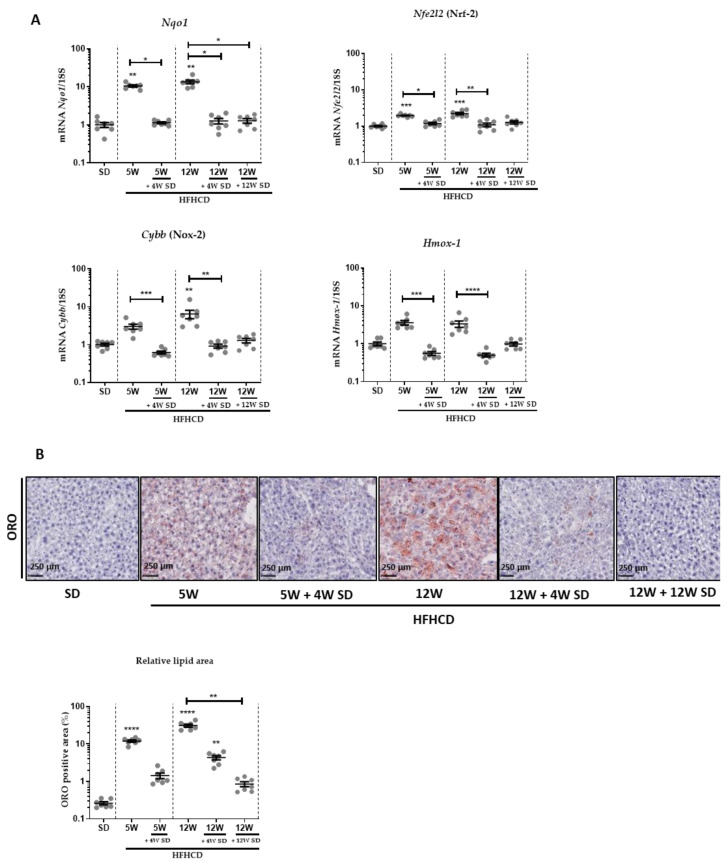
Dietary intervention reduced NASH-induced oxidative stress and steatosis. Six groups of seven male mice were given separate diet regimens of either a standard diet (SD) or a high-fat, high-cholesterol diet (HFHCD) for 5 or 12 weeks (W), with some groups returning to the SD on an additional period of 4 or 12 weeks. (**A**) Liver transcript expression of Nqo1, Nfe2l2 (Nrf-2), Cybb (Nox-2) and Hmox-1. (**B**) Oil red O (ORO) stained liver sections and quantification of ORO positive area (% area of lipid deposition). For all graphs, each dot represents an individual and error bars are expressed as means ± SEM (* *p* < 0.05, ** *p* < 0.01, *** *p* <0.001 and **** *p* <0.0001).

HFHCD feeding also resulted in hepatocyte ballooning and the recruitment of inflammatory cells, as evidenced in H&E-stained tissues (Figure 1C). Likewise, immune cell infiltration significantly declined when mice returned to an SD for 4 weeks (Figure 1C,D). Accordingly, the expression of liver transcripts of inflammatory markers like Tnf-α and Ccl2 significantly increased in proportion to the time of HFHCD (Figure 1E). When mice were fed again with the SD after 5 or 12 weeks of HFHCD, these mRNA amounts returned to levels similar to those present in healthy mice only fed an SD. Since oxidative stress plays a key role in the pathogenesis of NASH, the mRNA expression of genes like Nqo1, Nfe2l2 (Nrf-2), Cybb (Nox-2) and Hmox-1 involved in oxidative stress were followed (Figure 2A). Again, these markers of oxidative stress were significantly induced by 5 or 12 weeks of HFHCD, and effects reversed as early as 4 weeks back to an SD.

Further, hepatic steatosis was also assessed at all experimental stages by Oil Red O (ORO) staining that was quantified (Figure 2B). The lipid content of hepatocytes raised and worsened when HFHCD was prolonged. Once the mice were returned to the SD, the steatosis diminished drastically, but residual staining persisted.

### 3.2. HFHCD-Induced Fibrosis Is Partially Reversible

**Figure 3:**
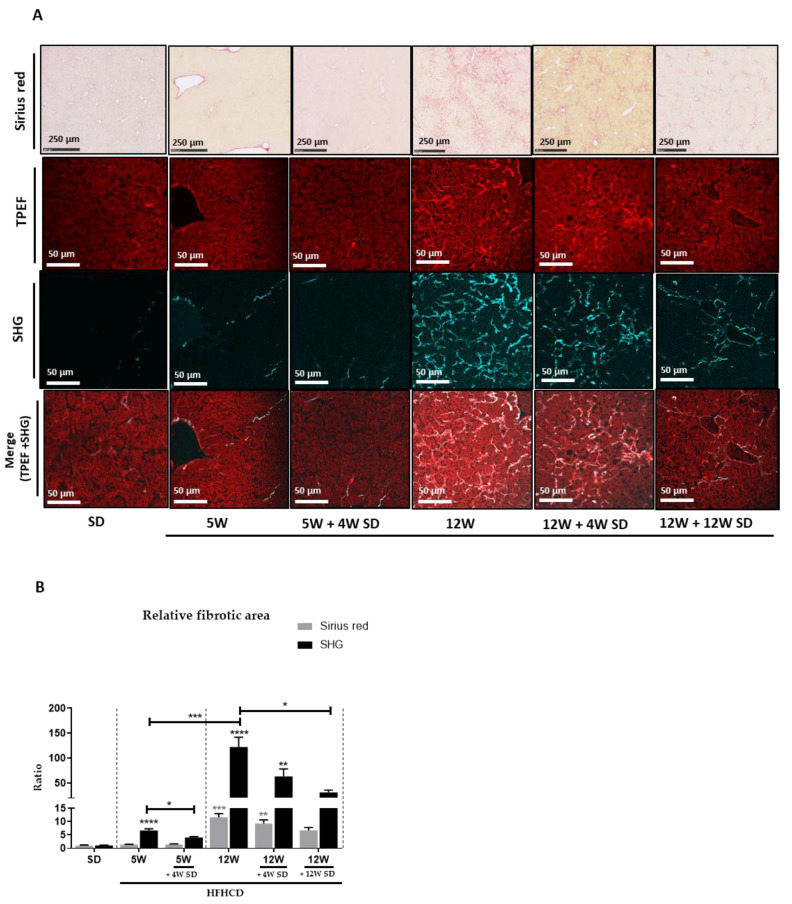
Dietary intervention significantly reduced NASH-induced fibrosis. Six groups of seven male mice were given separate diet regimens of either a standard diet (SD) or a high-fat, high-cholesterol diet (HFHCD) for 5 or 12 weeks (W), with some groups returning to the SD on an additional period of 4 or 12 weeks. (**A**) Liver sections either stained with Sirius red (upper panels) or multiphoton imaging by two-photon excitation fluorescence (TPEF; liver tissue auto-fluorescence; red; second line panels) and second-harmonic generation (SHG; collagen; blue; third line panels) and a merge of both (TPEF + SHG; lower panels). (**B**) Quantification of Sirius red and SHG signals; signal fold changes compared to the SD condition arbitrarily set at 1 for each approach. Statistical analysis was performed by applying non-parametric Kruskal-Wallis test (* *p* < 0.05, ** *p* < 0.01, *** *p* < 0.001 and **** *p* < 0.0001).

To quantify the level of fibrosis, a first analytical approach was used based on the classic labeling of collagen fibers using Sirius red (Figure 3A, upper panels). Signal assessment by measuring the percentage of stained area seemed to indicate that 5 weeks of HFHCD was insufficient to initiate significant fibrosis. However, significant fibrotic scarring was revealed in the livers of mice fed during 12 weeks with HFHCD. Thus, approximately 10% of the parenchymal tissue was replaced by connective tissue. By switching the mice to the SD, the scar rate gradually and slowly decreased at 4 then 12 weeks to reach 6% of the tissue surface. To complete the investigations on fibrosis in this mouse model, second-harmonic microscopy was used to visualize and quantify fibrillary collagen accumulating in the liver. The auto-fluorescence of the liver tissue, acquired by TPEF, allowed for the visualization of the hepatic parenchyma structure, while the SHG signal revealed the fibrillary components of ECM. At present, the greater sensitivity of this analytical method revealed early weak fibrosis already established after 5 weeks of HFHCD, which had gone unnoticed with the previous colorimetric staining. Thus, the SHG-detected signal, revealing fibrillary collagen around vessels and sinusoids, increased by more than 6 times and more than 120 times after, respectively, 5 and 12 weeks of HFHCD compared to healthy livers. Resuming the mice to an SD dropped the SHG signal significantly. Thus, depending on the length of the prior HFHCD period extending over 5 or 12 weeks, the amount of collagen detected by the SHG approach decreased by 1.6 or 2 times, respectively, after 4 weeks of SD. This decrease reached 4 times after 12 weeks of SD in the protocol where mice had previously been subjected to 12 weeks of HFHCD. Even if it was partial, a progressive reversion of the fibrosis was thus observed.

**Figure 4:**
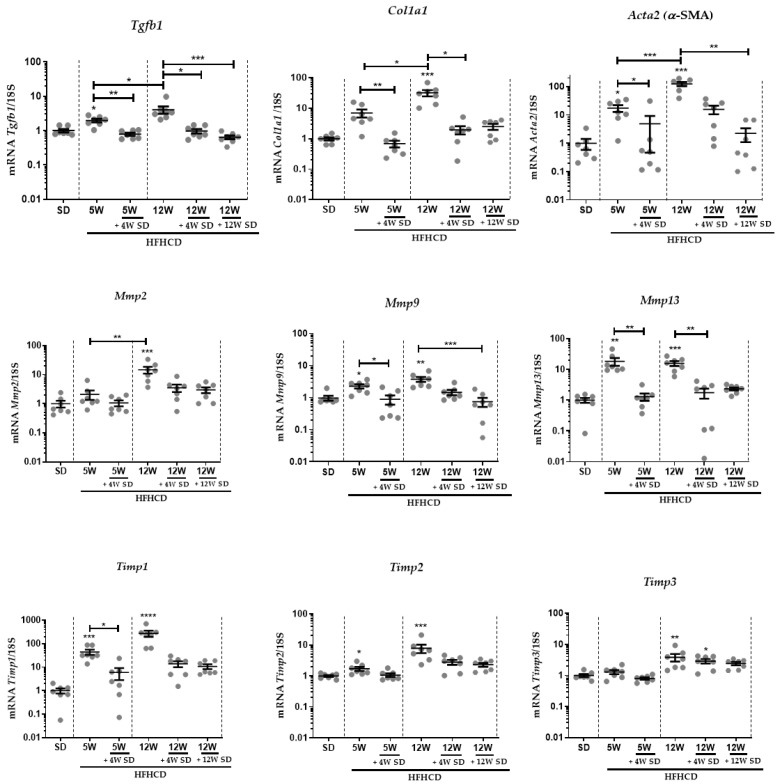
Liver transcript expression of genes involved in ECM deposition and degradation. Six groups of seven male mice were given separate diet regimens of either a standard diet (SD) or a high-fat, high-cholesterol diet (HFHCD) for 5 or 12 weeks (W), with some groups returning to the SD on an additional period of 4 or 12 weeks. Liver transcript expression of TGFB1, Col1a1, Acta2 (αSMA), Mmp2, Mmp9, Mmp13, Timp1, Timp2 and Timp3. For all graphs, each dot represents an individual, and error bars are expressed as means ± SEM (* *p* < 0.05, ** *p* < 0.01, *** *p* < 0.001 and **** *p* < 0.0001).

To complete these later approaches, carried out at the level of tissue architecture, transcript amounts of genes involved in ECM remodeling were quantified in the livers of mice engaged in each tested feeding protocol. Furthermore, levels of liver transcripts of genes indicative of liver fibrosis, such as transforming growth factor beta 1 (TGFB1), actin alpha 2 (Acta2) (αSMA) and collagen type I alpha 1 (Col1a1), were measured. The expression of these genes continued to rise with the increase in the duration of HFHCD feeding. However, switching to a standard diet resulted in significant a decrease in the expression of these genes. These changes were more pronounced at 12 weeks of standard diet feeding after 12 weeks of HFHCD feeding (Figure 4, upper panel). During the fibrosis process, other genes that are involved in ECM remodeling, such as matrix metalloproteinases (Mmp2, Mmp9 and Mmp13) and some of their regulators, tissue inhibitors of metalloproteinases (Timp1, Timp2 and Timp3), were thus chosen for analysis. Quantifications showed that similar variations, with induction rates specific to each gene, occurred for the different markers tested (Figure 4). With the exception of Mmp13, mRNA levels of other metalloproteases (Mmp2 and Mmp9) increased as the time on HFHCD was prolonged. As soon as mice had been re-fed with the SD for at least 4 weeks, all of these markers relapsed at rates close to those measured in healthy livers. Only the Timp3 mRNA started a moderate decrease that was slightly accentuated after 12 weeks of SD in the animals, which had previously been subjected to 12 weeks of HFHCD.

## 4. Discussion

Liver fibrosis results from chronic and persistent liver injury. The balance between ECM deposition and degradation normally prevailing in a healthy liver is deregulated. The scar tissue gradually expands when the activated HSCs increase the deposition of fibrous proteins, and when at the same time, the degradation of liver ECM is no longer ensured by MMPs, which are excessively inhibited by TIMPs [20,21,22,23]. Currently, there is no FDA-approved treatment for NASH patients [24,25,26], for which liver fibrosis remains the strongest predictor of mortality [27]. Human epidemiological studies showed that dietary changes and exercise ameliorate histological parameters, such as steatosis and ballooning in NASH [28,29]. However, there are conflicting reports on the effect of dietary intervention or exercise and bariatric surgery on the reversibility of fibrosis in NASH [25,29]. In murine models of NASH, data on the reversibility of advanced fibrosis are still sparse [30,31].

In the present study, mice were fed an HFHCD for 5 or 12 weeks, a treatment known to induce NASH, which leads to severe fibrosis [18]. In accordance with some other models of NASH (MCD, CDAA or WD) [14,15,16], when mice returned to a standard chow diet for a period of 4 or even 12 weeks, not only were the steatosis and inflammatory status of livers greatly improved but also markers of oxidative stress were normalized. Furthermore, the SD shift resulted in the cessation of new liver damage, as demonstrated by the basal level of serum transaminases and diminished hepatomegaly. The return of these clinical indicators to levels found in healthy livers was accompanied by a significant and progressive decrease in fibrosis. However, unlike the other symptoms, fibrosis reversed more slowly. Dietary intervention must be maintained over a long period to recover from fibrosis induced by HFHCD feeding, especially when damage levels reach high stages. Consistently, another independent study showed, in a group of mice with CDAA-induced NASH, that liver fibrosis persisted after dietary intervention over a 7-weeks recovery period [16].

High-fat non-cholesterol or high-cholesterol non-fat feedings were reported to cause simple steatosis in mice, while once combined, a high-fat and high-cholesterol diet resulted in a significant increase in liver inflammation, steatosis, hepatocytes ballooning, immune infiltration and fibrosis [32,33]. In humans, NASH patients have eating habits with a richer cholesterol intake [34]. In rodents, liver fibrosis induced by chronic administration of CCl4 or alcohol resolved completely within 4–7 weeks when CCl4 or alcohol exposure was, respectively, withdrawn [13,35]. Conversely, in our model of HFHCD-induced fibrotic NASH, fibrosis only gradually recovered after dietary intervention. Using the SHG approach to assess liver scarring, fibrosis was detected as early as 5 weeks of treatment, while it remained undetectable by the usual Sirius red staining. This SHG sensitivity allowed the detection of an early stage of fibrosis, which also took time to resolve when returning to a healthy diet. Interestingly, the hepatic mRNA levels of TIMPs reached after 12 weeks of HFHCD did not return to their basal level detected in healthy animals, even after a long period (12 weeks) on SD. This phenomenon could be a key element explaining the slowness of fibrosis reversion, especially since it has been shown that treatment based on anti-TIMP1 antibodies leads to an improvement in hepatic fibrosis induced by CCl4 in rodents [36]. Recently, a study showed in a mouse model that combining physical exercise with dietary modifications improved several clinical parameters, such as atherosclerosis, adipose tissue inflammation and even skeletal muscle function, but no additive effect was detected for fibrosis reversion, which remained partial even after 20 weeks [31].

## 5. Conclusions

Our results demonstrated that switching to a regular diet after cessation of the HFHCD quickly led to a disappearance of hepatomegaly, oxidative stress and hepatic damage, as well as a marked improvement in steatosis and inflammation, while fibrosis regressed only partially and slowly. This implies that NASH-associated pathological changes, including fibrosis, can be reversed by avoiding the factors that trigger NASH and by maintaining a standardized diet. Since the risks of liver-related morbidity and mortality are linked to fibrosis, pharmaceutical approaches designed to treat this symptom will most likely need to be combined with dietary intervention to potentiate them.

## Data Availability

Data is contained within the article or Supplementary Materials.

## Supplementary Materials

The following are available online at https://www.mdpi.com/article/10.3390/nu14020386/s1. Table S1: SD, HFHCD composition in proteins, carbohydrates and fats. Table S2: SD, HFHCD composition in fats. Table S3: Primer sequences. Figure S1: Schematic illustration of all treated groups. Figure S2: Switching to regular diet resulted in significant changes in body weight, liver damage and inflammation.

## References

[1] Powell E.E., Wong V.W., Rinella M. (2021). Non-alcoholic fatty liver disease. Lancet.

[2] Marchisello S., Di Pino A., Scicali R., Urbano F., Piro S., Purrello F., Rabuazzo A.M. (2019). Pathophysiological, Molecular and Therapeutic Issues of Nonalcoholic Fatty Liver Disease: An Overview. Int. J. Mol. Sci..

[3] Benedict M., Zhang X. (2017). Non-alcoholic fatty liver disease: An expanded review. World J. Hepatol..

[4] Luedde T., Kaplowitz N., Schwabe R.F. (2014). Cell death and cell death responses in liver disease: Mechanisms and clinical relevance. Gastroenterology.

[5] Goldberg D., Ditah I.C., Saeian K., Lalehzari M., Aronsohn A., Gorospe E.C., Charlton M. (2017). Changes in the Prevalence of Hepatitis C Virus Infection, Nonalcoholic Steatohepatitis, and Alcoholic Liver Disease Among Patients with Cirrhosis or Liver Failure on the Waitlist for Liver Transplantation. Gastroenterology.

[6] Buzzetti E., Pinzani M., Tsochatzis E.A. (2016). The multiple-hit pathogenesis of non-alcoholic fatty liver disease (NAFLD). Metab. Clin. Exp..

[7] Bedossa P., Paradis V. (2003). Liver extracellular matrix in health and disease. J. Pathol..

[8] Zbodakova O., Chalupsky K., Tureckova J., Sedlacek R. (2017). Metalloproteinases in liver fibrosis: Current insights. Met. Med..

[9] Caligiuri A., Gentilini A., Pastore M., Gitto S., Marra F. (2021). Cellular and Molecular Mechanisms Underlying Liver Fibrosis Regression. Cells.

[10] Rockey D.C. (2016). Liver Fibrosis Reversion After Suppression of Hepatitis B Virus. Clin. Liver Dis..

[11] Vilar-Gomez E., Martinez-Perez Y., Calzadilla-Bertot L., Torres-Gonzalez A., Gra-Oramas B., Gonzalez-Fabian L., Friedman S.L., Diago M., Romero-Gomez M. (2015). Weight Loss through Lifestyle Modification Significantly Reduces Features of Nonalcoholic Steatohepatitis. Gastroenterology.

[12] Caldwell S.H., Argo C.K. (2015). Reversing Advanced Hepatic Fibrosis in NASH: Clearly Possible, but Widely at Hand?. Dig. Dis. Sci..

[13] Iredale J.P., Benyon R.C., Pickering J., McCullen M., Northrop M., Pawley S., Hovell C., Arthur M.J. (1998). Mechanisms of spontaneous resolution of rat liver fibrosis. Hepatic stellate cell apoptosis and reduced hepatic expression of metalloproteinase inhibitors. J. Clin. Investig..

[14] Lytle K.A., Jump D.B. (2016). Is Western Diet-Induced Nonalcoholic Steatohepatitis in Ldlr-/- Mice Reversible?. PLoS ONE.

[15] Mu Y.P., Ogawa T., Kawada N. (2010). Reversibility of fibrosis, inflammation, and endoplasmic reticulum stress in the liver of rats fed a methionine-choline-deficient diet. Lab. Investig. J. Tech. Methods Pathol..

[16] Takeuchi-Yorimoto A., Noto T., Yamada A., Miyamae Y., Oishi Y., Matsumoto M. (2013). Persistent fibrosis in the liver of choline-deficient and iron-supplemented L-amino acid-defined diet-induced nonalcoholic steatohepatitis rat due to continuing oxidative stress after choline supplementation. Toxicol. Appl. Pharmacol..

[17] Benyon R.C., Iredale J.P. (2000). Is liver fibrosis reversible?. Gut.

[18] Vasseur P., Dion S., Filliol A., Genet V., Lucas-Clerc C., Jean-Philippe G., Silvain C., Lecron J.C., Piquet-Pellorce C., Samson M. (2017). Endogenous IL-33 has no effect on the progression of fibrosis during experimental steatohepatitis. Oncotarget.

[19] Simoes Eugénio M., Farooq M., Dion S., Devisme C., Raguenes-Nicol C., Piquet-Pellorce C., Samson M., Dimanche-Boitrel M.T., Le Seyec J. (2020). Hepatocellular Carcinoma Emergence in Diabetic Mice with Non-Alcoholic Steatohepatitis Depends on Diet and Is Delayed in Liver Exhibiting an Active Immune Response. Cancers.

[20] Han Y.P. (2006). Matrix metalloproteinases, the pros and cons, in liver fibrosis. J. Gastroenterol. Hepatol..

[21] Duarte S., Baber J., Fujii T., Coito A.J. (2015). Matrix metalloproteinases in liver injury, repair and fibrosis. Matrix Biol. J. Int. Soc. Matrix Biol..

[22] Giannandrea M., Parks W.C. (2014). Diverse functions of matrix metalloproteinases during fibrosis. Dis. Models Mech..

[23] Hemmann S., Graf J., Roderfeld M., Roeb E. (2007). Expression of MMPs and TIMPs in liver fibrosis-a systematic review with special emphasis on anti-fibrotic strategies. J. Hepatol..

[24] Sanyal A.J., Friedman S.L., McCullough A.J., Dimick-Santos L. (2015). Challenges and opportunities in drug and biomarker development for nonalcoholic steatohepatitis: Findings and recommendations from an American Association for the Study of Liver Diseases-U.S. Food and Drug Administration Joint Workshop. Hepatology.

[25] Younossi Z.M., Loomba R., Rinella M.E., Bugianesi E., Marchesini G., Neuschwander-Tetri B.A., Serfaty L., Negro F., Caldwell S.H., Ratziu V. (2018). Current and future therapeutic regimens for nonalcoholic fatty liver disease and nonalcoholic steatohepatitis. Hepatology.

[26] Sumida Y., Yoneda M. (2018). Current and future pharmacological therapies for NAFLD/NASH. J. Gastroenterol..

[27] Ekstedt M., Hagström H., Nasr P., Fredrikson M., Stål P., Kechagias S., Hultcrantz R. (2015). Fibrosis stage is the strongest predictor for disease-specific mortality in NAFLD after up to 33 years of follow-up. Hepatology.

[28] Romero-Gómez M., Zelber-Sagi S., Trenell M. (2017). Treatment of NAFLD with diet, physical activity and exercise. J. Hepatol..

[29] Promrat K., Kleiner D.E., Niemeier H.M., Jackvony E., Kearns M., Wands J.R., Fava J.L., Wing R.R. (2010). Randomized controlled trial testing the effects of weight loss on nonalcoholic steatohepatitis. Hepatology.

[30] Verbeek J., Spincemaille P., Vanhorebeek I., Van den Berghe G., Vander Elst I., Windmolders P., van Pelt J., van der Merwe S., Bedossa P., Nevens F. (2017). Dietary intervention, but not losartan, completely reverses non-alcoholic steatohepatitis in obese and insulin resistant mice. Lipids Health Dis..

[31] van den Hoek A.M., de Jong J., Worms N., van Nieuwkoop A., Voskuilen M., Menke A.L., Lek S., Caspers M.P.M., Verschuren L., Kleemann R. (2021). Diet and exercise reduce pre-existing NASH and fibrosis and have additional beneficial effects on the vasculature, adipose tissue and skeletal muscle via organ-crosstalk. Metab. Clin. Exp..

[32] Savard C., Tartaglione E.V., Kuver R., Haigh W.G., Farrell G.C., Subramanian S., Chait A., Yeh M.M., Quinn L.S., Ioannou G.N. (2013). Synergistic interaction of dietary cholesterol and dietary fat in inducing experimental steatohepatitis. Hepatology.

[33] Liang J.Q., Teoh N., Xu L., Pok S., Li X., Chu E.S.H., Chiu J., Dong L., Arfianti E., Haigh W.G. (2018). Dietary cholesterol promotes steatohepatitis related hepatocellular carcinoma through dysregulated metabolism and calcium signaling. Nat. Commun..

[34] Musso G., Gambino R., De Michieli F., Cassader M., Rizzetto M., Durazzo M., Fagà E., Silli B., Pagano G. (2003). Dietary habits and their relations to insulin resistance and postprandial lipemia in nonalcoholic steatohepatitis. Hepatology.

[35] Sun M., Kisseleva T. (2015). Reversibility of liver fibrosis. Clin. Res. Hepatol. Gastroenterol..

[36] Parsons C.J., Bradford B.U., Pan C.Q., Cheung E., Schauer M., Knorr A., Krebs B., Kraft S., Zahn S., Brocks B. (2004). Antifibrotic effects of a tissue inhibitor of metalloproteinase-1 antibody on established liver fibrosis in rats. Hepatology.

