# Supplementary material for: Switching to Regular Diet Partially Resolves Liver Fibrosis Induced by High-Fat, High-Cholesterol Diet in Mice
Source: Nutrients. 2022 Jan 17;14(2):386. doi: 10.3390/nu14020386 (PMC8778944; doi:10.3390/nu14020386)
Supplement: Supplementary file 1 [file nutrients-14-00386-s001.zip › nutrients-1521392-supplementary.pdf]

Diet composition:
Suppliers provided diet compositions are mentioned in Table S1 and S2. Table S1 depicts the energy density (Kcal/g) with energetic part of protein, carbohydrates and fat of the standard diet (SD) and the high-fat high-cholesterol diet (HFHCD). Table S2 depicts the composition in lipids (% by weight) of the standard diet (SD) and the high-fat high-cholesterol diet (HFHCD).

Table S1: SD, HFHCD composition in proteins, carbohydrates and fats

Diet composition	SD	HFHCD
Energy density	3 (Kcal/g)	6.5 (Kcal/g)
Protein	22%	6%
Carbohydrates	66%	8%
Fats	12%	86%

Table S2: SD, HFHCD composition in fats

Fats composition	SD	HFHCD
Total fats (%) by weight	4	61.9
Total saturated fatty acids	0.6	36.4
Total monounsaturated fatty acids	0.7	20.4
Total polyunsaturated fatty acids	2.1	2.1
Cholesterol	0	1.5

Table S3. Primer sequences.

**Mouse Gene**	**Forward primers**	**Reverse primers**
18S	5′-CGCCGCTAGAGGTGAAATTC-3′	5′-TTGGCAAATGCTTTCGCTC-3′
Tnf-α	5′-TAGCTCCCAGAAAAGCAAGC-3′	5′-TTTTCTGGAGGGAGATGTGG-3′
Ccl2	5′-TCCCAATGAGTAGGCTGGAG-3′	5′-TCTGGACCCATTCCTTCTTG-3′
Hmox-1	5′-AGGTACACATCCAAGCCGAGA-3′	5′-CATCACCAGCTTAAAGCCTTCT-3′
Cybb **(Nox-2)**	5′-AGTGCGTGTTGCTCGACAA-3′	5′-GCGGTGTGCAGTGCTATCAT-3′
Nfe2l2 **(Nrf-2)**	5′-TAGATGACCATGAGTCGCTTGC-3′	5′-GCCAAACTTGCTCCATGTCC-3′
Nqo1	5′-AGGATGGGAGGTACTCGAATC-3′	5′-AGGCGTCCTTCCTTATATGCTA-3′
Tgfb1	5′-CACCATCCATGACATGAACC-3′	5′-CAGAAGTTGGCATGGTAGCC-3′
Acta2 **(α-SMA)**	5′-GTCCCAGACATCAGGGAGTAA-3′	5′-TCGGATACTTCAGCGTCAGGA-3′
Col1a1	5′-GCTCCTGCTCCTCTTAGGG -3′	5′-GCAGAAAGCACAGCACTCG -3′
Timp1	5′- TTCCAGTAAGGCCTGTAGC-3′	5′-TTATGACCAGGTCCGAGTT -3′
Timp2	5′-CTGGGACACGCTTAGCATCA -3′	5′-GACAGCGAGTGATCTTGCAC -3′
Timp3	5′-GAAGCCTCTGAAAGTCTTTGTGG-3′	5′-ACATCTTGCCTTCATACACGC-3′
Mmp2	5′- ATGGTAAACAAGGCTTCATG-3′	5′-TTGAGAAGGATGGCAAGTAT -3′
Mmp9	5′-CATTCGCGTGGATAAGGAGT -3′	5′-TCACACGCCAGAAGAATTTG-3′
Mmp13	5′-ACACTGGCAAAAGCCATTTC-3′	5′-TTTTGGGATGCTTAGGGTTG-3′

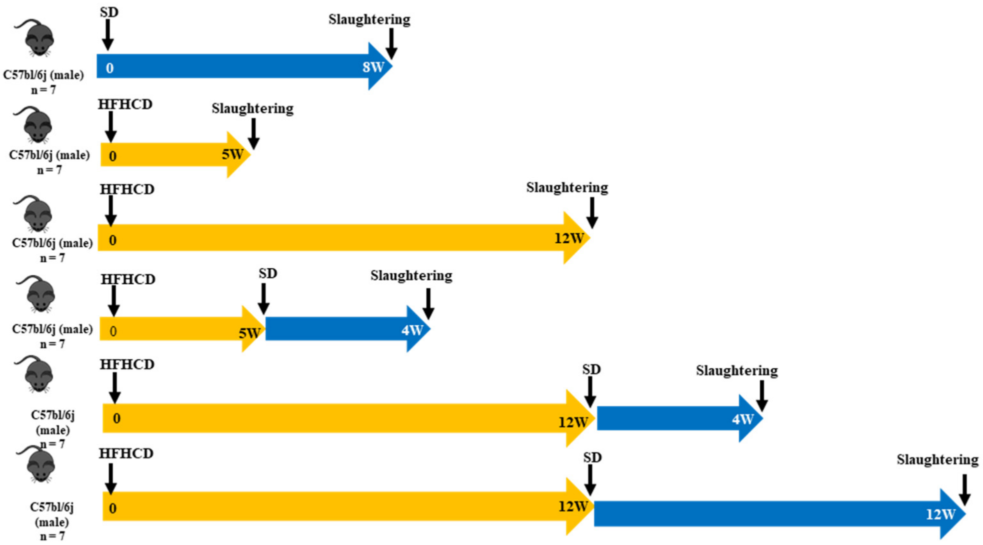

Figure S1: Schematic illustration of all treated groups.

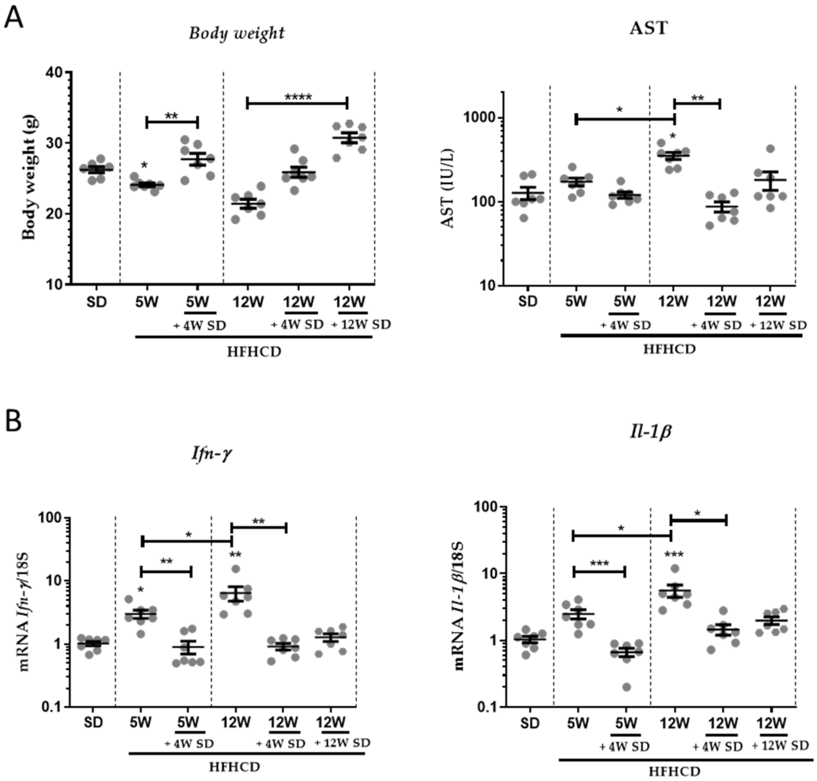

Figure S2: Switching to regular diet resulted in significant changes in body weight, liver damage and inflammation. Six groups of seven male mice were given separate diet regimens of either a standard diet (SD) or a high-fat high-cholesterol diet (HFHCD) for 5 or 12 weeks (W) with some groups returning to the SD for an additional period of 4 or 12 weeks. (A) Total body weight of each mice in grams and levels of AST in plasma. (B) Liver transcript expression of *Ifn-γ* and *Il-1β*. For all graphs, each dot represents an individual and errors bars are expressed as means ± SEM (**p* < 0.05, ** *p* < 0.01, *** *p* < 0.001 and **** *p* < 0.0001).
